# Ethical considerations for integrating multimodal computer perception and neurotechnology

**DOI:** 10.3389/fnhum.2024.1332451

**Published:** 2024-02-16

**Authors:** Meghan E. Hurley, Anika Sonig, John Herrington, Eric A. Storch, Gabriel Lázaro-Muñoz, Jennifer Blumenthal-Barby, Kristin Kostick-Quenet

**Affiliations:** ^1^Center for Medical Ethics and Health Policy, Baylor College of Medicine, Houston, TX, United States; ^2^Department of Child and Adolescent Psychiatry and Behavioral Sciences, Children’s Hospital of Philadelphia, Philadelphia, PA, United States; ^3^Department of Psychiatry and Behavioral Sciences, Baylor College of Medicine, Houston, TX, United States; ^4^Center for Bioethics, Harvard Medical School, Boston, MA, United States; ^5^Department of Psychiatry and Behavioral Sciences, Massachusetts General Hospital, Boston, MA, United States

**Keywords:** neuroethics, neural data, computer perception, digital phenotyping, affective computing, privacy, neurorights

## Abstract

**Background:**

Artificial intelligence (AI)-based computer perception technologies (e.g., digital phenotyping and affective computing) promise to transform clinical approaches to personalized care in psychiatry and beyond by offering more objective measures of emotional states and behavior, enabling precision treatment, diagnosis, and symptom monitoring. At the same time, passive and continuous nature by which they often collect data from patients in non-clinical settings raises ethical issues related to privacy and self-determination. Little is known about how such concerns may be exacerbated by the integration of neural data, as parallel advances in computer perception, AI, and neurotechnology enable new insights into subjective states. Here, we present findings from a multi-site NCATS-funded study of ethical considerations for translating computer perception into clinical care and contextualize them within the neuroethics and neurorights literatures.

**Methods:**

We conducted qualitative interviews with patients (*n* = 20), caregivers (*n* = 20), clinicians (*n* = 12), developers (*n* = 12), and clinician developers (*n* = 2) regarding their perspective toward using PC in clinical care. Transcripts were analyzed in MAXQDA using Thematic Content Analysis.

**Results:**

Stakeholder groups voiced concerns related to (1) perceived invasiveness of passive and continuous data collection in private settings; (2) data protection and security and the potential for negative downstream/future impacts on patients of unintended disclosure; and (3) ethical issues related to patients’ limited versus hyper awareness of passive and continuous data collection and monitoring. Clinicians and developers highlighted that these concerns may be exacerbated by the integration of neural data with other computer perception data.

**Discussion:**

Our findings suggest that the integration of neurotechnologies with existing computer perception technologies raises novel concerns around dignity-related and other harms (e.g., stigma, discrimination) that stem from data security threats and the growing potential for reidentification of sensitive data. Further, our findings suggest that patients’ awareness and preoccupation with feeling monitored via computer sensors ranges from hypo- to hyper-awareness, with either extreme accompanied by ethical concerns (consent vs. anxiety and preoccupation). These results highlight the need for systematic research into how best to implement these technologies into clinical care in ways that reduce disruption, maximize patient benefits, and mitigate long-term risks associated with the passive collection of sensitive emotional, behavioral and neural data.

## 1 Introduction

Computer perception (CP) technologies hold potential to revolutionize personalized care in psychiatry and other disciplines by improving diagnostics, symptom and treatment response monitoring, and even prediction of relapse, longitudinal shifts in symptom severity, or future disease development (Picard, 1997; Sheth et al., 2016; Barnett et al., 2018; Insel, 2018; McDonald et al., 2023). CP technologies include a range of approaches such as digital phenotyping, affective computing, computer vision and computational behavioral analysis which utilize algorithms to analyze passive and continuous behavioral data collected from patients outside of clinical contexts (i.e., “in the wild”) using wearables and other personal devices such as smart phones (Insel, 2014; Onnela and Rauch, 2016; Torous et al., 2021). These devices capture data that are often sensitive in nature, including but not limited to GPS location, facial movements, vocal acoustics, heart rate, accelerometry, and even social media usage, which can be analyzed on their own or in combination with established clinical measures and self-reports to reveal patterns in patients’ behavioral and emotional states (Sheikh et al., 2021). These approaches differ from remote patient monitoring (RPM) in that the range of collected digital metrics are intended to provide insights into the manifestations of psychiatric and behavioral conditions in daily life using continuous, objective measures, thereby reducing reliance on cross-sectional self-reports, which may burden patients and offer limited insights into patients’ subjective experiences and symptomology (D’Mello et al., 2018; Raugh et al., 2021; Saganowski et al., 2022; Charron et al., 2023). Excitement around these technologies has been tempered by practical limitations in understanding the clinical significance of these computational behavioral data due to challenges in accounting for their contextual and subjective significance (Zhao et al., 2019). Further, a growing literature has begun to draw attention to ethical implications, particularly privacy concerns and potential for negative impacts (e.g., perceived dehumanization) on clinical care (Martinez-Martin, 2022). While these concerns are emerging around computer perception more broadly, little attention has been paid to specific ethical considerations raised by the potential of integrating more conventional forms of computer perception (i.e., using wearables) with other data streams, particularly neural data, that have strong potential to automatically detect, understand and even influence subjective states (Smith et al., 2021).

These capacities are already expanding in the field of neurology, where computer perception is being used in tandem with neurotechnologies such as deep brain stimulation (DBS) to explore the psychosocial and behavioral correlates of neural activity data (Rainey and Erden, 2020). This new field of study is called “computational neuroethology,” or the science of quantifying naturalistic behaviors for understanding the brain (Datta et al., 2019). Investigations in this field range from animal studies to discover neuromarkers of animal behavior to studies with human subjects to explore neural markers of psychosocial and behavioral states, typically for the purposes of applied (rather than basic) clinical research (Torous et al., 2019). Examples include studies linking neural activity to vocal and sociobehavioral indicators of Parkinson’s disease (Smith et al., 2017); facial and vocal markers of schizophrenia (Xu et al., 2022), and psychosocial markers of major depressive disorder (Mundt et al., 2012), with a goal of identifying personalized treatment (e.g., stimulation) approaches based on patient-specific symptom constellations and neural patterns. These efforts expand the repertoire of biometric and behavioral markers guided by a hope that neural markers may provide more direct representations of internal states such as emotions, cognition or intentions (Sheth et al., 2022). Here, we report qualitative findings from our ongoing research into ethical considerations around the integration of computer perception technologies into clinical care, as well as insights from our previous work exploring neuroethical considerations for invasive neurotechnologies such as DBS (Muñoz et al., 2020; Zuk et al., 2020; Kostick-Quenet et al., 2023), to anticipate how concerns around computer perception technologies may be exacerbated by their expansion into areas of neurology and neuromodulation.

## 2 Background

Ethical concerns about the implementation of computer perception technologies largely revolve around potential violations of privacy and data security (Martinez-Martin and Kreitmair, 2018; Tomičić et al., 2022). Ethicists and clinicians alike recognize that the characteristically passive methods of data collection for these technologies, using wearables and personal devices such as smartphones, create ample opportunity for such violations (Segura Anaya et al., 2018). These technologies frequently collect vast amounts of individual data from individuals as they go about their daily routine at home, work, school or other private (and public) settings, offering clinicians a glimpse into patients’ behaviors, feelings, or mental states outside of traditional clinical settings. The sensitive nature of many of these data underscores the need for strong data protection (Mohr et al., 2020). Some have highlighted the importance of transparency, responsible data stewardship, and informed patient consent as ways to address privacy concerns (Martinez-Martin et al., 2021) and urge clinicians and researchers to be clear about what data are being collected and why, how data are being stored and who has access, and to disclose to patients the risks of patient (re)identification. Others have begun to catalogue and examine different data types utilized by computer perception technologies and their associated threats to patient privacy, acknowledging that not all forms of passive data collection raise privacy concerns to the same degree (Martinez-Martin et al., 2018; Connolly et al., 2021; Mulvenna et al., 2021). Important to note is that many of these ethical concerns about privacy and monitoring may be relevant more broadly across remote patient monitoring and general digital health markers; this article, however, focuses primarily on digital mental health indicators because of their direct implications for potentially revealing mental states. These implications raise ethical concerns (e.g., data sensitivity and privacy) distinct from physical data.

Concerns are also emerging around the perceived invasiveness of automatic and direct detection of patients’ emotions and mental states. One of the most unique purported capacities of novel computer perception technologies is the ability to automatically infer emotional, cognitive or mood states via biobehavioral measurements (Hammal and Cohn, 2018; Saganowski et al., 2022). Should this capacity be accurately and reliably realized, patients risk losing control and autonomy over the disclosure of their private, subjective experience. Importantly, the unique methods and capabilities of computer perception technologies may violate privacy both in the traditional sense (relating to data security and identification noted above) but also in a newer, deeper sense, violating what scholars now refer to as “mental privacy” (Wajnerman Paz, 2021; Farahany, 2023; Susser and Cabrera, 2023). These new types of threats have recently gained attention in the neuroethics literature, with scholars calling for “neurorights” (Yuste et al., 2017) to mental integrity, privacy and cognitive liberty (Bublitz, 2013; Shen, 2013; Ienca, 2017; Ienca and Andorno, 2017; Lavazza, 2018; Farahany, 2019; Jwa and Poldrack, 2022; Wajnerman Paz, 2022). These rights revolve around the notion that individuals should be free from influence or interference in their subjective experiences and should have agency over their neural data and any insights that may be obtained or inferred from them. Rapid advancements in neural “decoder” technologies, for example, can reconstruct and record continuous internal (unspoken) language from cortical semantic representations using functional magnetic resonance imaging (fMRI) (Tang et al., 2023). Similar advancements are underway using other neurotechnologies, with promising applications for conditions like locked-in syndrome (Kübler, 2020; Branco et al., 2023) and paralysis (Savage, 2018). These emerging technologies have demonstrated the growing potential for neural activity – in conjunction with advancements in AI – to directly reveal what people may be thinking or feeling.

Further, invasive neurostimulation tools like deep brain stimulation (DBS) (Lozano et al., 2019) and non-invasive neuromodulatory tools like transcranial magnetic stimulation (TMS) (Zhong et al., 2021) and other direct-to-consumer neural devices (Kreitmair, 2019) highlight the potential for not only detecting internal mental states but also for modulating them in targeted ways. The demonstrated efficacy and visible symptom reduction for many patients utilizing these technologies offer new hope for individuals suffering from certain refractory conditions such as depressive (Mayberg et al., 2005) and obsessive compulsive disorders (Abelson et al., 2005); however, they also raise questions about unwanted, external control over patients’ internal mental states. Qualitative studies suggest that certain stakeholders consider these capacities even more worrisome when stimulation is automated, such as with adaptive DBS (aDBS) (Goering et al., 2017; Outram et al., 2021; Kostick-Quenet et al., 2022; Merner et al., 2023) These “closed loop” systems not only record and analyze neural data but also respond to individual neural activity patterns in a feedback loop to automatically deliver personalized patterns of stimulation to the brain (Priori et al., 2021). Some patients and caregivers have questioned whether these capacities open the door to external manipulation of brain and behavioral states and/or unintended future uses of neural data (Kellmeyer, 2021).

As awareness of these concerns continues to grow, little is known about how advancements in neurotechnology, computer perception and particularly AI-based systems may combine to enable capacities for direct and automatic detection of subjective states. Further, knowledge gaps remain over whether stakeholders’ existing ethical concerns about computer perception technologies may be exacerbated by the potential integration of neural data. Here, we present insights from a study (R01TR004243) examining high priority concerns around computer perception technologies and considerations for their responsible translation into clinical care. Specifically, we provide stakeholder perspectives from interviews with clinicians, developers, patients, and caregivers on privacy, data security and disclosure, and monitoring of behavioral and emotional states using computer perception (via personal devices and wearables) and contextualize these concerns within the broader neuroethics literature to explore ethical implications of integrating neural data with more conventional computer perception data streams.

## 3 Materials and methods

This research was conducted as part of a larger, multi-site study exploring perspectives on risks and concerns around the integration of computer perception technologies into clinical care. The study is ongoing and involves a partnership with a “sister” study funded by the National Institute for Mental Health (R01MH125958) to validate computer perception tools designed to quantify digital biobehavioral markers of socio-emotional functioning. Respondents included adolescent research participants participating in this partner study and the caregivers of these participants, as well as clinicians and developers of computer perception tools recruited as part of our primary study, as described below.

### 3.1 Participants

Participants were members of four stakeholder groups: (1) research participants familiar with in-clinic passive monitoring and data collection, a transdiagnostic sample of adolescents (aged 12−17years) with primary diagnoses of autism, anxiety, obsessive-compulsive disorder (OCD), or depression (*n* = 20), (2) caregivers of these adolescents (*n* = 20), (3) clinicians with varying medical specialties and levels of familiarity with computer perception technologies (*n* = 12), (4) developers of these technologies (*n* = 12), and (5) clinician developers with both a background / current practice in medicine and expertise in the development of these technologies (*n* = 2) (Table 1). Recruitment and data collection occurred between January 2023 and August 2023. Caregivers were referred by study coordinators from the sister study and then contacted by a research assistant via phone or email to schedule an interview. Diagnostic presentations for all adolescents were confirmed by expert clinicians through their participation in the sister study, using standardized established clinical measures for assessing autism, anxiety, and depression. Clinicians and developers were recruited via our extensive professional networks within Baylor College of Medicine (BCM) and Children’s Hospital of Philadelphia (CHOP) and/or identified via literature review to find key players involved in developing and testing computer perception tools for use in clinical research and care.

**TABLE 1:** Demographics for interviewed adolescents and caregivers.

	Adolescents		Caregivers		Clinicians		Clinician Developers		Developers		TOTAL
	***n* = 20**	**Total 30%**	***n* = 20**	**Total 30%**	***n* = 12**	**Total 18%**	***n* = 2**	**Total 3%**	***n* = 12**	**Total 18%**	***n* = 66**
**Gender**											
Male	12	60%	2	10%	5	42%	1	50%	10	83%	**45%**
Female	8	40%	18	90%	7	58%	1	50%	2	17%	**55%**
**Ethnicity**											
American Indian or Alaska Native	0	0%	1	5%	0	0%	0	0%	0	0%	**2%**
Asian	1	5%	1	5%	3	25%	0	0%	0	0%	**8%**
AA/Black	5	25%	4	20%	0	0%	0	0%	0	0%	**14%**
Native Hawaiian or Other Pacific Islander	0	0%	0	0%	0	0%	0	0%	0	0%	**0%**
White	17	85%	15	75%	9	75%	2	100%	12	100%	**83%**
Hispanic or Latino	4	20%	2	10%	1	8%	0	0%	1	8%	**12%**
Not Hispanic or Latino	16	80%	18	90%	11	92%	2	100%	11	92%	**88%**
**Marital status**											
Married & living w/ spouse			13	65%							**65%**
Widowed			1	5%							**5%**
Divorced			4	20%							**20%**
Separated			1	5%							**5%**
Never Married			1	5%							**5%**
**Education**											
Below high school	7	35%									**35%**
Partial high school, no diploma	12	60%	0	0%							**60%**
High school graduate			0	0%							**0%**
Partial college			0	0%							**0%**
Trade school/Associate’s degree			2	11%							**11%**
Bachelor’s degree			10	47%							**47%**
Master’s degree			4	21%							**21%**
Doctoral degree			4	21%							**21%**
**Parental status**											
Biological parent			18	90%							**90%**
Step parent			0	0%							**0%**
Adoptive parent			2	10%							**10%**
OCD	4	20%									**20%**
Autism	5	25%									**25%**
ADHD	3	15%									**15%**
Anxiety	4	20%									**20%**
Tourettes	1	5%									**5%**
No clinical diagnosis	9	45%									**45%**
Average age	14.6 (s.d. 2.1)		47.2 (s.d. 6.1)		42.1 (s.d. 9.0)		37.0 (s.d. 4.2)		47.0 (s.d. 4.3)		

### 3.2 Data collection

Separate but parallel interview guides were developed for clinicians, developers, adolescents and caregivers, with the same constructs explored across all stakeholder groups. Constructs explored for the broader study aims included perceived benefits and concerns regarding integrating computer perception tools into clinical care, impacts on care, attitudes toward automatic and passive detection of emotional and behavioral states, perceived accuracy and potential for misinterpretation/-attribution/-classification of symptoms or conditions, clinical utility and actionability, data security and privacy concerns, potential for unintended uses, and perceived generalizability and potential for bias. These domains were chosen based on issues raised in the clinical and ethics literature (see Background) and with the guidance of experienced bioethicists (KK-Q) and child mental health experts (ES, JH). Specific domains explored for the purposes of this current sub-study included attitudes toward automatic and passive detection of emotional and behavioral states, data security and privacy concerns, clinical utility and significance of neural data, and potential for misinterpretation/-attribution of internal states. Initial drafts of the interview guides were piloted with two psychologists specializing in adolescent mental health, resulting in minor clarifications in wording. Adolescents and caregivers were also shown a brief video explaining computer perception technologies and how they work to ensure informed responses. The content and language of the explainer video were chosen to reflect approximately grade school to high school reading level. Concepts such as computer vision, digital phenotyping, and wearable technology were defined and explained in simplified terms using analogies such as viewing digital phenotypes as a “puzzle” made up of puzzle pieces (data points collected from wearable technologies) and listing examples of wearable technologies that adolescents and caregivers would likely be familiar with. Additionally, the explainer video was paused after the explanation of each key concept and takeaway so that adolescents and caregivers could ask follow-up questions and researchers could ensure their understanding of the concepts before proceeding to the interview questions. Interviews were conducted via a secure videoconferencing platform (Zoom for Healthcare) and lasted an average of ∼45 min. This study was reviewed and approved by the Baylor College of Medicine Institutional Review Board (Approval #H-52227), which also waived a requirement for written consent; thus, participants provided verbal consent.

### 3.3 Data analysis

Interviews were audio-recorded, transcribed verbatim, and analyzed using MAXQDA software. Led by a qualitative methods expert (KK-Q), team members (KK-Q, MH, AS) developed a codebook to identify thematic patterns in stakeholder responses to questions addressing the topics above. Each interview was coded by merging work from at least two separate coders to reduce interpretability bias and enhance reliability. We identified patterns in the data using Thematic Content Analysis in MAXQDA to inductively identify themes by progressively abstracting relevant quotes, a process that entails creating distinct code outputs. For the purpose of this manuscript, code outputs were derived both from relevant codes in the code book developed as part of our larger study aims, and from a lexical search in MAXQDA, involving the identification of every mention of eight neural-related terms seen in each of the 66 stakeholder interviews. The eight terms included in the lexical search – brain, neuro, neural, DBS, fMRI, EEG, internal state, and mental state – were chosen to identify and explore each mention of forms of neural data for analysis. We chose to conduct this lexical search based on the observation that stakeholders often spontaneously raised (without being asked) perspectives regarding the perceived value of neural data as a potential complement (or vice versa) to insights from psychosocial and behavioral data collection via computer perception tools, despite that these topics were not explored directly via our interview questions or anticipated by our code book. Our analytical process involved reading every quotation to which a given code was attributed, paraphrasing each quotation (primary abstraction) and further identifying which constructs were addressed by each quotation (secondary abstraction). To reduce interpretability bias, abstractions were validated by at least one other member of the research team. The results presented below represent primary concerns / themes raised and discussed by stakeholders in these interviews related to privacy and data security concerns and specific concerns raised by the integration of neural data with other common computer perception data in healthcare.

## 4 Results

Three main themes emerged from our analysis. All four stakeholder groups voiced concerns about (1) perceived invasiveness of passive and continuous data collection in private settings; (2) data protection and security and the potential for negative downstream/future impacts on patients of unintended disclosure; and (3) ethical issues related to patients’ limited versus hyper awareness of passive and continuous data collection and monitoring. These concerns were especially prevalent among patients and caregivers (see Table 2). Clinicians and developers in particular highlighted how (4) the above concerns may be exacerbated by the integration of neural data with other computer perception data.

**TABLE 2:** Prevalence of concerns about passive monitoring among stakeholder groups.

	Adolescents (*n* = 20)		Caregivers (*n* = 20)		Clinicians (*n* = 12)		Developers (*n* = 12)		Clinician Developers (*n* = 2)		TOTAL (*n* = 66)	
Theme 1 - Perceived Invasiveness of Passive and Continuous Monitoring	16	80%	13	65%	10	83%	5	42%	1	50%	44	67%
Theme 2 - Uncertainties around Data Protection and Security	11	55%	17	85%	12	100%	9	75%	2	100%	54	82%
Theme 3 - Awareness of Passive and Continuous Data Collection and Monitoring	6	30%	7	35%	6	50%	4	33%	1	50%	24	37%

Frequencies were calculated by counting all unique respondents who raised / endorsed each of the theme concerns. Frequencies reflect only reported information and are not intended to suggest any level of statistical significance.

### 4.1 Perceived invasiveness of passive and continuous data collection in private settings

Interviews with patients, caregivers, and clinicians revealed a range of sentiments about the impact of passive data collection on patients’ feelings of privacy, particularly under specific circumstances, such as in intimate and private settings or situations involving highly personal or vulnerable moments (e.g., using the restroom or bathing, engaging in intimate/sexual interactions with partners, and participating in personal conversations with friends, family, or significant others). For example, one patient noted that he would not want audio recordings taken of him in particular because he wouldn’t want to *“feel like (he’s) being watched all the time by someone” (P_15)*. Some caregivers echoed this point, admitting that while a certain amount of tracking of a child’s whereabouts or activities is acceptable to ensure a child’s safety, the act of constant and multi-modal data collection is *“overkill” (CG_04)*, suggesting they perceive it as excessive. One caregiver described this type of automatic data collection as *“personal intrusiveness” (CG_11)* while another emphasized that patients *“deserve some privacy” (CG_04)*. Another caregiver raised concerns emotion detection being taken out of context, and further, felt that emotion detection taken *in* context would be *“way too invasive”* (CG_07). Moreover, some caregivers and patients noted the importance of preserving opportunities for private thoughts and solitude. One patient described the desire to occasionally turn off data collection devices to be alone—*“there might be times where I really want to just turn off the location, and be off the grid for a little while” (P_09)*—while another identified self-conscious thoughts or feelings as something they would prefer to keep private (P_05) and a third noted worry about passive data being shared with others when they are dealing with something personal (P_14).

Importantly, clinicians emphasized that for some patients, continuous data collection may feel too invasive for them to accept in their clinical care. One clinician explained:

*“There are going to be people who say, “This does feel like a violation of my privacy. I want to be able to control what I say and what I share and what I communicate to others about my feelings. I’m not going to do that.” Those people just won’t do it.” (C_06)*

### 4.2 Uncertainties around data protection, security and the potential for negative impacts of unintended disclosure

Several clinicians and developers pointed out that the novel and investigational nature of computer perception technologies and their integration with other technologies (e.g., machine learning) may leave passively collected data vulnerable to unanticipated, unintended uses if improperly protected. As one developer pointed out,

*“You just record somebody’s voice and then who knows what you can do with that in the future. It’s kind of like if I gave my genome sample a decade ago, who knows what you could say about what I can smell, what susceptibility I have to a given disease or disorder down the line? That information’s only going to become more and more clear in the future, much more actionable, as people are able to pull together different modalities.” (D_07)*

A clinician made a similar comparison to blood samples:

*“It’s not always clear what the future use of data collected today is going to be… people have stored blood samples to use with future tools that don’t exist yet to gain insights that we can’t even conceptualize right now.” (C_04)*

Another clinician highlighted that these concerns are especially pronounced computer perception data are indefinitely maintained and linked to individuals, with limited data protections:

*“The vast majority of (data collection systems) are, basically, Hoover vacuums that just suck everything into the cloud and are forever tied to an identity. So, when we think about the impact that that might have on healthcare, there are multiple different (and) so many weird scenarios that could come from that, because these things may exist into perpetuity. I don’t particularly like most of the privacy laws, because I don’t think they go … far enough.” (C_07*)

When asked how they feel about automatic behavioral and emotional data collection, one patient articulated similar concerns about data linked to them existing in perpetuity:

*“… They’re going to use that (data) for whatever and (it’s like) the Internet, when you (post) something (and) it’s stuck forever (there)” (P_05)*

### 4.3 Limited versus hyper awareness of passive and continuous data collection and monitoring

#### 4.3.1 Limited awareness and consent

Some clinicians emphasized the difficulties of consent and disclosure with patients being monitored at all times. One clinician explained that consenting to such extensive and continuous data collection means that patients cannot always anticipate what private life events they will have documented:

*“I think that the idea of passive data collection, when someone may not be actively choosing, “Yes, I want these researchers to know about this,” without the context of what’s going on… You experience a particularly stressful event, you experience something out of the norm or within the norm for you, but you don’t yet know what you’re consenting to have sort of recorded.” (CD_02)*

Indeed, when considering the life events that may be detected or personal information that may be inferred from passive and continuous monitoring, one caregiver remarked:

*“Well what if I didn’t want to tell you that?” It kind of crosses over when you can detect my emotion. I might not want to tell you that me and hubby had an argument last night, that’s none of your business. So that kind of weirded me out a little bit” (CG_11)*

Another clinician, reflecting on consent and continuous monitoring, speculated about the potential inability for patients to participate in and consent to voluntary disclosure, knowing that their data – and inferences from them gathered by computer perception technologies– would be received by a physician regardless:

*“I do think that consent is important here. So, I do wonder that if a patient who was agreeing to the use of this technology, if they would talk to their mental health professional differently if they knew that their true thoughts and feelings were always revealable, if there wouldn’t be any reason to conceal or to not be fully disclosing in therapy sessions, et cetera, because they knew that I was going to find out anyway.” (C_06)*

One developer also warned against the potential risk or harm of using data to derive direct insights into people’s inner thoughts and feelings without even having to ask them. Using data such as heart rate or blood flow in an example scenario, they explained:

*“(If I’m) analyzing video data so that I know… what your heart rate is based on blood flow through your face…and based on the heart rate, I’m making a guess about how you’re feeling or what you’re thinking, and you don’t know I’m doing that. Aside from the ethics of gathering that information… if I make a decision based on that or I accuse you of having a different motive or thinking or feeling a certain different way, then I’ve left you out of the conversation (and all the) many different things that could be going on through your head and your heart right now.” (D_07)*

#### 4.3.2 Hyper-awareness and preoccupation

All four stakeholder groups emphasized concerns about how feeling monitored could negatively impact self-perception and patient behavior, with potentially negative impacts on clinical care, including the clinician-patient relationship. For example, some respondents suggested that awareness of being monitored could lead patients to change their behaviors in ways that may be counterproductive to clinical progress.

For example, one patient explained how passive monitoring could impact her presentations of self – both to others and to herself:

*“It might make me kind of self-conscious about it and… I don’t know, it might affect the way I act, because if I’m trying to act a certain way to act for these devices, it might be changing me, which I don’t know if that’s a good thing… I think I would focus on how I’m feeling more than maybe I should be… And especially if I’m trying to hide something. I don’t think it’s a good idea for me to have to even try and hide it from myself. If you’re trying to hide it from other people, it would be kind of annoying, (or) if you had to hide it from even yourself, because there’s monitors on you.” (P_14)*

Caregivers also noted the possibility of unintended behavioral effects from interacting with computer perception technologies. Two caregivers shared:

*“I don’t know (if continuous, passive data collection) would cause her to change and not be her. (She) might sense that, “Oh, all of this has been picked up. Do I have to start acting a certain way? Do I need to start saying certain things?”” (CG_16)*

*“…if she’s aware (these data are being) gathered, I don’t even know if it would be honest because I think it would change the way that she would act” (CG_07)*

One caregiver was also concerned about the impact that hyper-awareness of passive and continuous monitoring may have on her adolescent’s mental health:

*“(Anxiety) might be a factor, an added factor, a stress factor to her already existing condition, and knowing that someone actually monitors her or that she’s being monitored in the most intimate, troubling situations.*

### 4.4 Imminence of integrating neural data with other forms of computer perception

Clinicians and developers suggest that integrating neural data with existing modes of computer perception may enhance the capacity to directly and automatically reveal information about patients’ emotional states. However, many pointed out certain limitations, particularly the lack of specificity and contextual information provided by certain forms of neural activity. For example, two clinicians explained:

*“FMRI does not get… the context piece (so) that you can see changes relative to context. And context is often a very personal and private thing. We don’t use FMRI very much in clinical practice because it’s not terribly actionable.” (C_04)*

*“We’re seeing portable EEG starting to come into (computer perception) work. But EEG is not as specific, so it’s not always as useful as you would like it to be.” (C_04)*

Another clinician focused on the limitations of neural data for indicating disorder, emphasizing that brain activity alone cannot and should not be used to inform diagnosis:

*“When you’re looking at brain rhythms, you’re looking at some sort of dysfunctional neural circuitry (which) is not specifically the jurisdiction of one disorder versus another. Nor is it sufficient to actually make diagnostic claims. So, when you take your brain signal and you use it exclusively to derive someone’s disorder, you’re actually changing from a Diagnostic and Statistics Manual disorder to a very specific brain circuitry disorder. And those are not the same things… I don’t think a brain recording should be used as a diagnostic criteria… Because that’s not how they (diagnostic criteria) were conceived, or what they were meant for originally, when the DSM was created.”(C_10)*

However, others were more optimistic and pointed to the potential diagnostic utility of neural data in the future. One developer said:

*“(Neural signatures are) definitely not ready for a diagnostic tool. But I think we’re laying the groundwork to move toward that point.” (D_10)*

Another developer suggested that this promise is elevated when neural data are combined with other data types:

*(Retinal blood vessels) actually have direct connections with how neurons develop in the brain, and as bipolar is partly a thought disorder, there are some physiological things you can pick up. So, there’s some great evidence around that. Then there is some evidence around EEG… If you really take a hard look… their accuracy is not going to exceed 70. Now, our psychiatrist and our team is going to say, “That is an insufficient performance for a psychiatrist like me to be conclusive, beyond what that might already observe. So, I’m not going to necessarily make a different clinical decision based on 70% accuracy.” However… if you would have a dataset that combines retinal scanning, EEG and blood based biomarkers…our hypothesis is that you can get into the high 90s percent. Now, that becomes clinically actionable.” (D_01)*

Another developer emphasized that, if certain implementation barriers could be overcome, integrating neural data with other computer perception technologies could provide a “remarkable” method for gaining insights into the brain:

*“If brain imaging were a little bit less expensive, more convenient, and more accessible, that could be a remarkable way of doing different types of digital phenotyping, activity-connectivity, whatever it is with the brain. You can (could) tell a lot about activity-connectivity and even morphology of a brain” (D_07)*

Others suggest that these capacities have already arrived:

*In terms of perceptual computing, I believe a lot of functional neurosurgery is actually going to be moving toward the direction of data acquisition, from either internal or external (sources)… due to Medtronic’s advent of actually having the ability to record and stimulate, as well as NeuroPace’s ability to record and stimulate. I think we’re going to be moving toward that direction. (C_10)*

Some clinicians emphasized that this capacity raises serious privacy concerns:

*“At its current level, I don’t think (neural data is) something that needs to be held extremely private… (recording a single band is) not going to give you a ton of granularity to what a person’s doing, thinking, etc.… it’s hard to really make a strong connection between the two of those. That being said, with whatever iteration a device is going to (have) down the line, the question becomes how much data are you getting, and at which point does it start to tell you a lot about what the person’s doing and thinking. Once you’re able to take a higher amount of data and actually deduce what that person was doing or thinking at that time, with some sort of reasonable accuracy… it becomes a very important prerogative to make sure that that data does not get outside of where a person intentionally wants it to go. Because in 10°years, you very easily could design a device that has recording channels… And via deep learning or AI, you can figure out what the person is most likely doing at any given time. And if someone were to, say, grab that data and download someone’s recording over the past month, you could deduce what they were doing over the course of the month… If we get to the point where you can deduce someone’s internal state, it becomes an immense breach of privacy.” (C_10)*

One clinician emphasized that monitoring brain activity can be even more invasive than other biobehavioral data indicating emotions:

*“…Emotion recognition… I think that’s invasive, but I think that potentially going into someone’s brain activity is even more invasive because people can have a poker face. We don’t always show what we’re feeling, or we can even fake out…But theoretically, if you could monitor someone’s brain activity, then you could know their deepest, darkest secrets that they’re trying to not let you know. That’s extremely intrusive. So, I think we have to be really, really careful about how that’s used.” (C_03)*

Moreover, the same clinician expressed particular concern about the integrating neural data and computer perception technologies with neuromodulatory tools that record and analyze neural data and can adjust stimulation parameters for patients in real time:

*“Using technology to monitor brain activity and even to change brain activity… it’s a tool and it depends on how it’s used… (and) who’s using it. I feel like that’s the ultimate breach, potentially, of privacy, to know what’s going on in our brains. The ultimate control over people could be to control their brain activity. So, I think that’s an extremely powerful tool, and we need a societal debate about how that would be used, an open ethical debate.” (C_03)*

## 5 Discussion

Stakeholders interviewed in our study conveyed a collective concern about the perceived invasiveness of passive and continuous data collection using computer perception devices, and worried about what may be detected about them (or their loved ones) in private, personal or intimate moments. These concerns confirm those raised in the literature addressing computer perception technologies, largely focused on privacy concerns related to collecting data that reveal individuals’ geographical location, call logs, voice dynamics, and other sensitive information (Onnela and Rauch, 2016; Fuller et al., 2017; Martinez-Martin and Kreitmair, 2018; Torous et al., 2019; Mulvenna et al., 2021). What is lacking in the extant literature is an exploration of how the range of data modalities collected using computer perception devices is rapidly expanding to include other data types, especially neural data, which are being explored in parallel for their capacity to directly and automatically detect emotional and cognitive states. The existing literature does not address whether the integration of these new modalities may exacerbate existing concerns raised around computer perception technologies, or whether their integration raises novel, unanticipated concerns that must be proactively addressed. Below, we argue that the integration of neural data with existing computer perception data indeed raises novel concerns related to self-determination and control over one’s destiny.

Responses from clinicians and developers suggest that privacy concerns are likely to be exacerbated by integrating neural data with more conventional computer perception data from wearables. The concern is that the synthesis of these data types could potentially provide a direct window into an individual’s thoughts, emotions and motivations, often in real time. This concern stems from the fact that, while certain types of neural data (e.g., intracranial neurophysiological measurements in isolation) may not be directly interpretable on their own, recent advancements suggest they may become increasingly interpretable and meaningful when contextualized by the rich situational and behavioral data captured by wearables (Smith et al., 2017; Datta et al., 2019). To appreciate this point, it is worth acknowledging the growing capacity of neural data on their own, even without integration of other data streams or self-reports, to provide direct insights into cognitive, emotional and intentional states. Recent studies suggest that it is possible to infer visual content of mental processing (Wen et al., 2018), imagined handwriting (Willett et al., 2021), or covert (internal) speech (Pawar and Dhage, 2020) from neural data (Reardon, 2023; Tang et al., 2023). Some scholars (Miller, 2010) rightfully caution that the ability to examine, measure and even manipulate these psychological phenomena does not indicate that we are any closer to understanding their nature or phenomenology (e.g., what thoughts are made of) or how the brain implements psychological phenomena via observable physiological or electrical processes. However, the aforementioned studies suggest that scientists (and industry) are getting closer to identifying the neural and physiological *associates* of psychological phenomena in ways that increasingly enable assessments of the presence or absence of these phenomena or shifts across conditions or time, as well as their manipulation. These capacities will likely continue to expand, enabled by parallel advancements in neurotechnology and AI – in particular, generative AI and large language models (LLMs) – with numerous benefits on the horizon for patients suffering from both physical and psychiatric symptoms that limit their capacity to effectively express thoughts and emotions. Direct detection of internal states may help these individuals achieve greater communication and social connectedness.

Emerging research demonstrates that these potentials may be enhanced by multimodal approaches. Methods like deep digital phenotyping (DPP), for example, have the capacity to illuminate the clinical and biobehavioral significance of previously indecipherable neural data by identifying patterned associations with physiological, digital, and other biometric data. Combining data from multiple modalities has been shown to reveal information about subjective perceptions (e.g., visual imagery and face perception) (Chang and Tsao, 2017), intentional states (e.g., motor plans; imagined speech) (Akbari et al., 2018), and affect (Sani et al., 2018). Integrating neural data into these data streams can help to elucidate how the brain behaves across different conditions “in the wild” as well as in response to targeted stimulation. To date, the most granular neural information comes in the form of intracranial neurophysiological data using recording-capable DBS devices or from electrocorticography strips placed on the brain’s surface (Rich and Wallis, 2017; Leszczyński et al., 2020). These mechanisms have demonstrated potential to elucidate personalized “neural signatures” with a high degree of specificity, improving localization accuracy and helping to identify brain regions that may play a role in specific cognitive functions, offering generalizable insights about emotion and behavior. Synchronizing these data with other passive collected measures and intermittent self-reports in non-clinical settings may help to identify neural signatures associated with fluctuations in daily functioning and to contextualize heterogenous responses of the brain. Monitoring and understanding these shifts in functioning can help clinical teams to respond to urgent care needs and to plan personalized treatments approaches.

### 5.1 Considerations related to informed consent and privacy

Patients consenting to neural DDP may be exposing personal and private information that they may not want revealed or may be subjectively unable to foresee. Further, they may not intuitively appreciate what sorts of inferences may be drawn about their current or future mental states or diagnostic status. As noted earlier, researchers are actively trying to detect and classify illness using computer perception, typically involving the use of algorithms to search for behavior and symptom constellations. Some research further points to the potential of using passively collected data to *predict* mental states using probabilistic generative models (Sukei et al., 2021). Neural data is poised to play an increasingly important role in these predictions, for the reasons discussed above (Kato et al., 2022).

Upholding patient privacy and protection of these data should thus be a primary priority. To date, computational representations of emotion and behavior do not receive any higher forms of data protection than other protected health information regulated by the Health Insurance Portability and Accountability Act (HIPAA) (United States Congress, 1996) or the EU General Data Protection Regulation (2016, 2023). Ongoing debates informing the recent U.S. Executive Order on the Safe, Secure, and Trustworthy Development and Use of Artificial Intelligence (Federal Register, 2023) as well as the European Commission’s AI Act (European Parliament, 2023) do address the particular sensitivity of biometric data and their capacity to reveal personal identifiers and their potential to be used in ways that are unrelated or even orthogonal to patient care. These debates acknowledge the growing capacity to infer meaningful information from even miniscule quantities of highly granular biometric data (e.g., 3s of a voice recording), highlighting the potential for reidentification and misuse of biometric data (Kröger et al., 2020). These concerns are only heightened when biometric data becomes multimodal, and when each data type on its own, let alone in tandem, offer opportunities to gain insights into individuals’ thoughts, emotions and motivations that may not be used for patients’ direct benefits. To the extent these data fall into the hands of third parties or bad actors who may use them in ways that harm patients, patients could suffer long-term risks that remain difficult to identify at the time of consent, and difficult to appreciate when data is being collected automatically, passively, and often outside an individual’s conscious (or constant) awareness. This concern extends to direct-to-consumer (DTC) neurotechnologies, given their capacity to collect neural data that consumers may not be aware of or may not have explicitly consented to (Ienca et al., 2018; Coates McCall and Wexler, 2020). Ensuring patients understand and consent to the collection of neural data is especially important for DTC technologies, considering these data are not typically considered “health data” and therefore receive less strict data protections than health data covered by HIPAA regulations, such as those collected by research-grade devices in the context of clinical research or care (Kreitmair, 2019). These gaps in data protection are made more complex by enduring uncertainties around health data ownership, given the multiple stakeholder groups that aid in its collection, storage, management and stewardship, which complicate understandings about what various entities are permitted to do with neural data (e.g., buy, sell, exchange). Outside of the consumer space, research groups such as those in the BRAIN Initiative, the National Institutes of Health and the National Institutes of Mental Health are encouraged and even mandated to share neural data with the research community in order to promote reuse of neural data in pursuit of new research directions and scientific discoveries and to minimize cost and waste around new data acquisition (Rahimzadeh et al., 2023). These well-intentioned policies are accompanied by an important tradeoff, particularly the risks that open data sharing introduces for data subjects (research participants) who are placed at greater risk for reidentification as capacities for data fusion and triangulation continue to expand.

Data security risks are even more crucial given that inferences from neural data – in combination with other biometric, genomic, and sociobehavioral data – may be used to make assumptions or generalizations not just about individuals but also about groups, and for purposes (e.g., political; social; economic; legal) unrelated to clinical care. Even de-identified data are at risk of being re-identified and used to make inferences about individuals and social groups (Price and Cohen, 2019). As neural data becomes more interpretable and available through the proliferation of research-grade and/or direct-to-consumer neurotechnologies, rapid advancements in AI may enable identification of patterns in neural activity across individuals, communities or even groups defined according to observed associative patterns (i.e., empirically-defined or “latent” groups that may not map onto social or community affiliations). In addition to re-identification risks, the identification of neural signatures associated with cognitive functioning, or stigmatized behaviors such as impulsivity (Aharoni et al., 2013), violence (Poldrack et al., 2018; Kolla et al., 2023), or even criminality (Poldrack et al., 2018) may open the door to matching individuals’ neural signatures (with some degree of probability) with broader patterns of neural activity observed across certain social groups. Proponents have argued that this kind of “AI neuroprediction” offers a way to increase the accuracy of violence risk assessment and to identify possible interventions to reduce the likelihood for criminal recidivism or other stigmatized behavior (Tortora et al., 2020). Others point out that matching individuals on the basis of neural signatures is an extreme and undesirable use of these technological advancements and will likely lead to further stigma and negative impacts on already marginalized groups. These risks are exacerbated by challenges in disentangling racial and institutionalized bias embedded into certain algorithms that may amplify incorrect assumptions drawn about certain groups and perpetuate negative stereotypes and prejudice.

Such potential misuses of neurotechnology and computer perception may threaten mental integrity, cognitive liberty, and self-determination (autonomy in determining one’s own destiny) (Farahany, 2023). These topics are currently receiving international attention and in need of further ethical discussion (Blumenthal-Barby, 2022; Ienca et al., 2022), and further research is needed to ensure that patient and caregiver education about benefits and risks at the time of consent account for potential downstream consequences. Expertise in patient communication will be required in order to effectively draw attention to concerns that may be perceived as hypothetical or abstract without unduly discouraging participation and engagement with technologies that may have justifiable benefits or value tradeoffs. Involvement from clinicians will be pivotal for ensuring that patient education and informed consent approaches are responsive to patient and caregiver concerns about perceived invasiveness and potential negative impacts from experiences with continuous data collection and monitoring. Further research is needed to identify patients’ and caregivers’ distinct informational needs for appreciating the benefits and risks of consenting to the collection and various uses of neural data. Greater consensus is also needed around what clinicians, neurotechnology developers, as well as policymakers and regulators feel that patients need to know in order to make informed decisions about the integration of neural data with other forms of computer perception, given the potentially sensitive inferences that may be drawn – both now and as technologies advance into the future.

### 5.2 Potential future impacts of hyper awareness of passive monitoring

Interestingly, our results highlight that while certain ethical dilemmas are generated by limited (i.e., “hypo”-) awareness of and consent to passive and continuous monitoring, others are generated by *hyper* awareness of the same. Some patients (familiar with in-clinic passive monitoring and data collection) said they would likely feel overly aware, preoccupied with or fixated on the fact of being constantly monitored or “surveilled” outside of the clinic. Some patients said they would likely feel self-conscious of their behaviors and, pressured by the knowledge that their clinicians or caregivers may have access to their passively collected data, might feel an urge to “hide certain behaviors” or change them to “act a certain way for the device.” Respondents who voiced these concerns about self-presentation during the potential appeared to fall into two categories: (1) patients who may hide or mask certain (true or authentic) mental states, thoughts or behaviors; and/or (2) patients who may attempt to change and alter certain mental states, thoughts, or behaviors, or produce (i.e., try to simulate or fake) certain thoughts or behaviors that they perceive to be “right” or desirable (either by themselves or their perceived understanding of what constitutes “right” or “good” behavior from their caregivers (if minors), clinicians, or other social or external influences). In some cases, such attempts could lead to the development of emotional or cognitive defenses that may be unproductive or even impede clinical progress by not providing clinicians with a full, accurate picture of an individual’s emotion state, and by extension, their health and well-being. For computer perception technologies to be utilized successfully in clinical care (e.g., for diagnostic purposes, symptom tracking, or treatment response prediction), they must be able to pick up on accurate, real-time data from patients in the context of their habitual settings and activities. However, “masking” could limit the accuracy, relevance and utility of collected data to inform clinical impressions and decision-making.

Beyond clinical utility, patients themselves may be harmed by the long-term consequences of persisting tendencies to suppress emotion or create self-enforced barriers to behaving authentically, potentially resulting in enduring developmental changes to emotion, cognition, behavior or personality that are patient-led rather than guided and supported by clinicians (Gross and Levenson, 1997). Especially for patients with conditions like OCD or post-traumatic stress disorder, with symptoms that involve certain thought, memory, or emotional suppression (Shipherd and Beck, 2005; Sinha and Chakrabarti, 2022), this additional self-directed managing of expression and formation or suppression of thoughts and emotions could negatively impact mental health. While these responses may be uncommon among patients, individuals with certain conditions or with certain dispositions or psychosocial characteristics may be particularly motivated to mask or neutralize their thoughts, motivations or behaviors, with potentially negative downstream consequences.

These responses may be magnified in cases where computer sensors capture neural activity, as neural activity is often viewed as [and some scholars argue are (Mecacci and Haselager, 2019)] more direct correlates of cognitive and affective states. Evidence from the neuroethics and aDBS literature suggests that certain patients receiving aDBS report uncertainties over whether their aDBS device is “controlling” or facilitating their actions, behaviors, or emotions (Muñoz et al., 2020; Zuk et al., 2020; Kostick-Quenet et al., 2023). As monitoring and modulation typically happen outside of conscious awareness, patients with aDBS may struggle with concerns of behavioral or emotional inauthenticity, uncertain whether their thoughts and behaviors are their own or computer-generated or -influenced. These impressions may inhibit individuals’ perceived freedom to act in accordance with their genuine self, limiting their perceived ability to choose how to behave and who to become. They may also negatively impact individuals’ perceived ability to self-determinedly pursue an “open future,” a term referring to the set of moral rights individuals (especially minors) possess to determine their own life choices before they are determined by others (Feinberg, 2014).

### 5.3 Mitigating risks and empowering patients

We suggest two potential avenues to explore to ensure that computer perception technologies utilizing neural data are responsibly translated into clinical care: (1) Identify and mitigate ways in which passive and continuous monitoring may harm patient development and self-perception and (2) Assist patients in feeling empowered rather than controlled by their health data. First, clinicians (and clinical researchers) employing computer perception technologies (including but not limited to those integrating neural data) could establish and encourage open, patient-clinician dialogue in the earliest stages of deploying these technologies, inviting patients to voice their concerns and questions, and giving clinicians the opportunity to educate patients about the intended purposes and capacities of these technologies. Clinicians should draw on their professional expertise and understandings of how a patient’s particular anxieties (related or unrelated to their condition), preoccupations or personal experiences may act as indications or counterindications for the use of certain types of computer perception tools, at certain times, or in certain scenarios. This clinical groundwork should be a standard prerequisite for the use of computer perception technologies in clinical care, helping to build trust and amplify patient voices in the earliest implementation stages. Urgent research is needed to inform best practices for personalizing computer perception approaches in ways that minimize disruption and maximize benefits of care.

Patients should also be invited to provide ongoing feedback on their experiences throughout the full period during which computer perception technologies are being used in their care. Forums for eliciting patient feedback should be strategized in advance and tailored to individuals, depending on the specificities of their condition, orientation toward technology, preferences and dispositions, and other factors that remain unexplored. Further research is also needed into how to disclose ongoing or summary insights from algorithms that process data from computer sensors. To what extent, and with what frequency, should patients receive feedback from these technologies? Would results be better delivered as notifications, summaries, or explained by clinicians or healthcare professionals specializing in patient communication? Should approaches to disclosure vary from patient to patient, by disorder type, or be determined after some period of empirical observation of clinical, emotional or behavioral reactions? A critical step toward mitigating the risks presented in this paper is to systematically identify concrete strategies by which patients’ voices and lived experiences are kept at the center of clinical care, acknowledging the value of patients ahead of the data, rather than the other way around.

## 6 Limitations

Our results only reflect the perspectives of individuals we interviewed and may thus have limited generalizability. Further, many of the reported perspectives come from adolescents, who may have outlooks and concerns that are distinct from those of adults. However, given that adult caregivers and other stakeholders expressed many of the same concerns – even about the potential for “masking” — suggests that these concerns may not be unique to adolescents. Additionally, despite our efforts to inform and educate adolescents and caregivers on computer perception technologies and related concepts imperative for understanding them, the explainer video that we provided to these stakeholder groups before asking for their perspective on the ethical and practical implications of these technologies was not formally validated before its use. We thus relied on caregivers and adolescents to be candid regarding their understanding of the concepts in the video so that these misunderstandings could be remedied with further explanation before the interview process.

## 7 Conclusion

Privacy concerns have been raised around the use of computer perception technologies in healthcare, and in parallel, around neurotechnologies that use computer sensors to capture (and in many cases, therapeutically respond to) neural activity. This paper contributes to this discussion by offering empirical insights into how stakeholders perceive threats to privacy introduced by computer perception, perceive these threats to be exacerbated by the integration of neural with multimodal data streams, and forecast the potential negative near- and longer-term impacts of these privacy concerns on patient care and well-being. Our findings suggest that the integration of neurotechnologies with existing computer perception technologies raises novel concerns about dignity-related harms (e.g., stigma, discrimination) that stem from data security threats and the growing potential for reidentification of sensitive data. Further, our findings suggest that patients’ awareness and preoccupation with feeling monitored via computer sensors ranges from hypo- to hyper-awareness, with either extreme accompanied by ethical concerns (consent vs. anxiety and preoccupation). These results highlight the need for systematic research into how best to implement these technologies into clinical care in ways that reduce disruption, maximize patient benefits, and mitigate long-term risks associated with the passive collection of sensitive emotional, behavioral and neural data. Continued collaboration among stakeholders, including patients and caregivers, clinicians, developers and researchers of neuro- and computer perception technologies will be crucial for understanding and anticipating the capacities, limitations and clinical impacts of these technologies. Moving forward, it will also be important for funding organizations to prioritize research proposals led by multidisciplinary teams, ideally with embedded ethicists, to ensure these collaborations.

## Data availability statement

The datasets presented in this article are not readily available because full datasets must remain unavailable in order to ensure de-identification of interview participants. Requests to access the datasets should be directed to kristin.kostick@bcm.edu.

## Ethics statement

The studies involving humans were approved by the Baylor College of Medicine Institutional Review Board. The studies were conducted in accordance with the local legislation and institutional requirements. The ethics committee/institutional review board waived the requirement of written informed consent for participation from the participants or the participants’ legal guardians/next of kin because research procedures (interviews, de-identification of transcripts and storage on secure servers) involved minimal risk to participating stakeholders. Verbal consent was obtained from each research participant before beginning interviews.

## Author contributions

MH: Conceptualization, Formal analysis, Investigation, Project administration, Writing – original draft, Writing – review & editing. AS: Investigation, Project administration, Writing – review & editing, Visualization. JH: Conceptualization, Funding acquisition, Methodology, Supervision, Writing – review & editing. ES: Conceptualization, Funding acquisition, Methodology, Supervision, Writing – review & editing. GL-M: Supervision, Writing – review & editing. JB-B: Supervision, Writing – review & editing. KK-Q: Conceptualization, Formal analysis, Funding acquisition, Investigation, Methodology, Project administration, Resources, Supervision, Visualization, Writing – original draft, Writing – review & editing.
